# Predictors of depression and suicidality among children and adolescents living with HIV and AIDS

**DOI:** 10.4102/sajpsychiatry.v31i0.2440

**Published:** 2025-09-04

**Authors:** Umar B. Musami, Jibril Abdulmalik, Victor C. Onyencho, Yesiru A. Kareem, Choja A. Oduaran, Abdurrahman Ashiru, Abdu W. Ibrahim

**Affiliations:** 1 Department of Mental Health, Faculty of Clinical Sciences, University of Maiduguri, Maiduguri, Nigeria; 2 Department of Medical Services, Faculty of Clinical Sciences, Federal Neuropsychiatric Hospital, Maiduguri, Nigeria; 3 Department of Psychiatry, Faculty of Clinical Sciences, University of Ibadan, Ibadan, Nigeria; 4 Asido Foundation, Ibadan, Nigeria; 5 Community Psychosocial Research, Faculty of Health Sciences, North-West University, Potchefstroom, South Africa; 6 Department of Clinical Services, Neuropsychiatric Hospital, Aro, Abeokuta, Nigeria

**Keywords:** Adolescents, AIDS, Children, Depression, HIV, Nigeria, Suicide

## Abstract

**Background:**

Human immunodeficiency virus (HIV) and acquired immunodeficiency syndrome (AIDS) and affective disorders often coexist, leading to suboptimal health outcomes. Poor management of this comorbidity can result in decreased medication adherence, increased hospitalisations and diminished quality of life.

**Aim:**

To determine the predictors and correlates of depression and suicidality among children and adolescents living with HIV and AIDS in Maiduguri, Nigeria.

**Setting:**

This study was conducted at the antiretroviral therapy (ART) clinic of the University of Maiduguri Teaching Hospital, Nigeria.

**Methods:**

A cross-sectional study was conducted to recruit 160 children and adolescents with HIV and AIDS who are attending ART clinics. Participants were selected using a systematic random sampling approach. The Schedule for Affective Disorders and Schizophrenia for School-Aged Children Present and Lifetime Version (K-SADS-PL), which is both a screening and diagnostic instrument, was administered to identify those with depression and suicidality.

**Results:**

Suicidality and depression rates were found to be 30.6% and 45.0%, respectively. Frequent hospital admissions and medication non-adherence were significantly associated with both depression and suicidality.

**Conclusion:**

This study highlights the significant mental health burden among children and adolescents living with HIV and AIDS. The association between frequent hospitalisations, medication non-adherence and emotional disorders emphasises the need for integrated mental health services within HIV and AIDS care.

**Contribution:**

This study offers insightful information about the mental health situation of children and adolescents living with HIV and AIDS (CALWHA), contributing to a deeper understanding of their unique needs and informing the development of targeted interventions.

## Introduction

In adolescents and children living with human immunodeficiency virus (HIV), depression and suicidality are significant comorbidities that have been associated with higher morbidity and mortality, as well as accelerated disease progression.^1^ The HIV pandemic continues to pose significant challenges to global health, particularly in terms of its effect on the mental health of children, adolescents and families.^2,3^ Despite tremendous progress in HIV prevention and treatment, millions of people continue to receive new diagnoses every year. In the year 2022, 39 million people were living with HIV worldwide, of which 2.6 million were children between the ages of 0–19 years.^4^ Sub-Saharan Africa continues to be the region most affected by HIV.^5^ According to the World Health Organization (WHO), in 2022, Nigeria was second only to South Africa, with about 3.4 million people living with HIV.^6^

Children associated with HIV and acquired immunodeficiency syndrome (AIDS) can be categorised into three: (1) children living with HIV and AIDS; (2) children affected by HIV and AIDS and (3) children infected and affected by HIV and AIDS. Children living with HIV and AIDS are a group of children who acquired HIV, usually through mother-to-child transmission during pregnancy, childbirth or breastfeeding. Children impacted by HIV and AIDS include those who live in families where the disease is present, as well as vulnerable or orphaned children who have lost one or both of their parents to the illness, even though they are not afflicted with the virus. Lastly, children infected and affected by HIV and AIDS are those children who are both living with HIV and AIDS and are affected by the disease.^7.8,9^ Children associated with HIV and AIDS face extreme physical, psychological and social challenges.^10^ They are physiologically more susceptible to opportunistic infections, growth retardation and delayed puberty as a result of immunosuppression.^11^ Many have psychological problems such as sadness, anxiety or suicidality, as well as stigma and discrimination.^12^

Previous studies have emphasised the relationship between low CD4 counts, treatment duration and psychiatric complications such as HIV-associated neurocognitive disorders, depression and suicidality.^13,14,15^ CD4+ T cell counts are an important immunological marker in children and adolescents, particularly in the context of HIV infection. Understanding age-related variations and factors influencing CD4+ T cell counts is essential for accurate interpretation and clinical decision-making. Scholarly research is needed to refine further the understanding of CD4+ T cell dynamics and their role in immune health throughout childhood and adolescence. The WHO defines immunosuppression in individuals older than 5 years as a CD4+ T lymphocyte count below 500 cells/mm^3^, indicative of a compromised immune status.^16,17^

The asymptomatic WHO stages (I and II) role in emotional disorders among people living with HIV and AIDS has been widely researched. A previous study found no significant association between asymptomatic HIV infection (WHO Stage I) and depression or anxiety.^18^ Another study found that individuals with asymptomatic HIV infection (WHO Stage I) did not have higher levels of emotional distress compared to HIV-negative individuals.^19^ In respect of Stage II, a study reported that asymptomatic HIV infection with moderate immune suppression (WHO Stage III) was not associated with depression.^20^ On WHO Stages III and IV and mental illness, the research found that individuals with asymptomatic HIV infection (WHO Stage III) had higher rates of depression and anxiety compared to those with asymptomatic HIV infection (WHO Stage I).^18^ Similar studies found that asymptomatic HIV-positive individuals (WHO Stage IV) had higher levels of emotional distress compared to those with asymptomatic HIV infection (WHO Stage I).^19^

Mental health has been significantly impacted by HIV and AIDS.^21^ In Nigeria, a cross-sectional study of teenagers with HIV was conducted among 201 teenagers. The study found depressive episodes and suicidality to be 17% and 45%, respectively.^1^ Children and teenagers who attend antiretroviral therapy (ART) teen clubs in Malawi participated in a study. Majority (73%) of children and adolescents had emotional and behavioural issues. Being female was a significant risk factor for a high score. Also, being raised by a single parent had a significant impact on the mental health status of the children.^21^ The most prevalent mental health problem among individuals living with HIV and AIDS in Nigeria, according to a scoping review of mental health interventions for this population, was depression (67%) followed by depression co-occurring with other mental health issues (50%).^3^

Numerous issues that affect children in sub-Saharan Africa, including child labour, physical punishment, domestic abuse and orphanhood, may have an impact on their mental health.^22^ According to a report, adults and children living with HIV have more adverse childhood experiences than the general population, especially in sub-Saharan Africa.^23^ Children and adolescents living with HIV and AIDS experience a variety of psychological and emotional issues that make them more vulnerable to depression and suicidality. Many of these children are bullied or excluded in school by their communities. This action further aggravates their feelings of loneliness and despair because of stigma.^24^ Inadequately managed HIV disclosure to children and adolescents often results in confusion, rage and emotional distress.^25^ This hinders their ability to comprehend the condition and ultimately places them at greater risk for depressive symptoms and suicidality.

Managing a chronic condition that requires regular medication, physician appointments and frequent hospitalisation may be emotionally demanding, with issues including ART adherence, ART side effects and pill fatigue leading to feelings of pessimism.^26^ Bogale et al. (2024) reported that suicidal ideation and attempts among HIV and AIDS patients in Ethiopia were 20% and 11%, respectively.^27^ This could be attributed to various factors associated with individual differences. In addition, this group of children experienced stigma and shame, especially among their peers, and this subsequently affected their self-esteem negatively. Moreover HIV-related impairments such as memory and attention problems generally affect their daily function.^28,29^

Healthcare systems in resource-limited settings, such as Northeastern Nigeria, confront major challenges in delivering mental health assistance to children and adolescents living with HIV and AIDS (CALWHA). Many healthcare facilities suffer from a serious scarcity of competent mental health specialists, making it difficult to recognise and treat depression and suicidality in CALWHA.^30^ Human immunodeficiency virus treatment services in many regions of Nigeria primarily focus on ART treatment and physical health rather than the psychological aspects of it, leading to cases of depression and suicidality remaining undiagnosed and untreated.^31^ Several systemic issues at the policy level affect mental health care access for CALWHA. For instance, Nigeria’s national HIV and AIDS response lacks adequate mental health integration, and the absence of policies on depression and suicidality in CALWHA limits essential psychological care and eventually leads to fragmented care.^31,32^

Despite the recognition of the psychosocial burden of HIV infection since the advent of the epidemic, the impact on young people has received minimal attention.^33^ Previous studies found that most patients experience emotional disorders such as anxiety, depression and suicidality at some point during their HIV infection.^34,35,36^ Compared to the general population, CALWHA experience higher rates of depression and suicidality.^21^ Furthermore, available literature reveals a paucity of published information on depression and suicidality among CALWHA in Nigeria, particularly in the northeast region.^3,37^ The presence of emotional disorders has significant implications for the management of HIV and AIDS in children and adolescents.^38^ Studies have shown that emotional disorders are associated with poor adherence to medication and hospital follow-up visits, decreased levels of immunity, recurrent hospitalisation, increased cost of care and poor quality of life.^39,40,41^ However, a high index of suspicion is often lacking regarding the identification and management of emotional disorders among CALWHA, which could be partly because of low levels of knowledge of the prevalence and related factors of these disorders.^42,43^ Therefore, the findings of this study will build on the existing literature as regards to the management of depression and suicidality in HIV. Lastly, there is a dearth of studies on the prevalence and relationship of depression and suicidality with clinical and laboratory characteristics among CALWHA in Maiduguri, Northeastern Nigeria. The study by Adeyemo and colleagues which was conducted in Southern Nigeria, mainly focused on the effects of adverse childhood experiences on depression and suicidality among adolescents living with HIV.^1^

Generally, this study aims to assess depression and suicidality among CALWHA attending ART clinics at the University of Maiduguri Teaching Hospital. The specific objectives are to describe the prevalence and relationship of depression and suicidality with clinical and laboratory characteristics in CALWHA. Moreover, to describe the influence of sociodemographic factors on depression and suicidality in CALWHA when gender is controlled for. On this note, the following questions were raised: (1) What is the prevalence of depression and suicidality among CALWHA, and what are the clinical and laboratory characteristics? (2) What are the clinical and laboratory predictors of depression and suicidality among CALWHA? (3) To what extent do sociodemographic factors influence depression and suicidality among CALWHA when gender is controlled for?

## Research methods and design

This cross-sectional design study was conducted at the paediatric and adult ART clinics at the University of Maiduguri Teaching Hospital, Maiduguri. It is a tertiary healthcare facility in northeastern Nigeria that provides specialist and general healthcare services to patients, as well as training for all categories of health workers. The paediatric and adult clinics are funded by the AIDS Prevention Initiative in Nigeria (APIN). The hospital services patients mainly from the six states in the northeast geopolitical zone of Nigeria and other parts of the country, as well as from the neighbouring countries of Chad, Niger and Cameroon. The city has a population of about 4 098 391, according to the 2006 census figures.

### Study population and sampling strategy

This study was extracted from a larger comparative study among 320 children and adolescents with HIV and sickle cell disease (SCD), out of which 160 were CALWHA. In the context of this current study, only the 160 participants with HIV and AIDS were extracted from the original study. The Kirkwood formula (Equation 1) was used to calculate the sample size,^44^


$$n={\left(\text{Z}\alpha +\text{Z}1-\beta \right)}^{2}\left[{\text{P}}_{1}(1-{\text{P}}_{1})+{\text{P}}_{2}(1-{\text{P}}_{2})\right]÷{\left({\text{P}}_{1}-{\text{P}}_{2}\right)}^{2}$$ [Eqn 1]

where Zα =standard normal deviate corresponding to 5% level of significance = 1.96; Z1–β = standard normal deviate corresponding to a power of 80% = 0.84; P_1_ = the prevalence of psychiatric morbidity among children and adolescents receiving ART = 48%^45^; P_2_ = the prevalence of depression among adolescents with SCD is reported to be 12.5%^46^; P_1_–P_2_ = although from the aforesaid values of P_1_ and P_2_, the difference will be 35.5%; however, for this study, the minimum difference to be detected was conservatively taken to be 15%. This assumption was taken in order to increase the power and to get a larger sample size; while *n* = minimum sample size in the two groups, *n* = (1.96 + 0.84)^2^ (0.48[1−0.48] + 0.12[1−0.125]) ÷ (0.15)^2^ = 125 (in each group). However, to correct for the non-response rate, assuming an anticipated response rate of 80%, the estimated sample size is increased to 156 in each group, for a total of 312 respondents. This was rounded up to 160 in each group, making a total of 320 respondents. A systematic random sampling technique with a sampling interval of 4 was employed to select the participants. The study population was made up of 160 CALWHA, of which 83 were males (51.9%) and 77 were females (48.1%).

### Inclusion criteria

- CALWHA between the ages of 6 years and 18 years who assented to participate and whose parents provided informed consent.
- CALWHA who had been diagnosed for at least 6 months and had been attending the clinics for at least 6 months.
- Able to communicate fluently in English or Hausa.

### Exclusion criteria

- Children and adolescents who were too ill to be interviewed.
- CALWHA with a history of psychiatric disorder prior to the diagnosis of HIV infection.
- CALWHA with a history of ot her chronic medical conditions.

### Study instruments

Study instruments included a pre-designed questionnaire for sociodemographic data and a clinical proforma for relevant clinical information extracted from patient case notes. The participants’ psychiatric conditions, including depression and suicidality, were evaluated using the Schedule for Affective Disorders and Schizophrenia for School-Aged Children Present and Lifetime Version (K-SADS-PL). The semi-structured K-SADS-PL interview was developed by Puig-Antich and Chambers in 1978 to assess the mental health of children.^47,48^ It assesses cases of psychopathology from the past and present, and is designed for interviewing both the parents and children. It has been adapted to the Diagnostic and Statistical Manual of Mental Disorders, Fifth Edition (DSM-5) diagnostic criterion.^47,49^ The K-SADS-PL includes three components: an introductory interview (demographic, health and other background information), a screening interview (82 symptoms related to 20 diagnostic areas) and five diagnostic areas – (1) affective disorders, (2) psychotic disorders, (3) anxiety disorders, (4) disruptive behavioural disorders and (5) substance abuse, tic disorders, eating disorders, and elimination disorders (enuresis, encopresis).^47^ In the K-SADS-PL depression screen, a score of 2 (threshold) on any core symptom such as depressed mood, anhedonia or irritability would necessitate administration of the full depression supplement. In the supplement, a diagnosis of major depression requires at least 5 of 9 symptoms rated at threshold (score of 2), including either depressed mood or anhedonia, lasting at least 2 weeks with clinically significant impairment.^47^ Also, in the K-SADS-PL suicidality screen, endorsement of any suicidal thoughts, plans or behaviours – even at a threshold score of 1 (subthreshold) – would require the full suicidality supplement. In the supplement, each domain (e.g., passive ideation, active ideation, plans and attempts) is rated for present and lifetime on a 0–2 scale, with clinical concerns and risk level determined based on threshold symptoms (score of 2) and intent, planning or behaviour.^47^ The K-SADS-PL has a strong inter-rater reliability of 0.75, excellent test-retest reliability (κ > 0.77) and good construct validity and criterion validity, as demonstrated by its associations with other measures of psychopathology using DSM-5 diagnostic criteria.^49,50^ This instrument has been used locally in paediatric care settings in Nigeria.^51,52^

### Study procedure

We conducted this study with the assistance of three resident doctors: one from the paediatrics department and two registrars in psychiatry. The resident doctors had at least 2 years of work experience. The lead researchers and the research assistants were trained on how to administer the study instruments, and practical sessions in the form of role plays were also conducted. All the children and adolescents attending the paediatric ART clinic were directed to the research assistants from the paediatrics department, who screened and recruited the clients. Participants and their caretakers were briefed on the study’s objectives and methodology. The parents or caregivers gave their informed consent, and the children gave their assent. Afterwards, parents or caregivers who had provided informed consent and the participants who had given assent were interviewed. Although, the interview was mainly for CALWHA, corroborative information was only sought from the parents or guardians when the main participant (children) could not provide comprehensive information in line with the interview guide. Therefore, there was no delineation between the children and parents or guardians regarding the information extracted. The interviews were conducted in the consulting rooms of the paediatric ART clinic of the University of Maiduguri Teaching Hospital. The rooms guarantee privacy and have functional air conditioning systems and comfortable seating arrangements. The lead investigator and the two psychiatry registrars administered the questionnaires. Only codes were used to identify the respondents to maintain confidentiality. The duration of the study was 6 months. The interview took place only on Tuesdays and Fridays, which are their normal clinic days. We spent an average of 1 h interviewing each participant. Participants with a positive score for either depression or suicidality were further interviewed using the diagnostic aspect of the K-SADS-PL of the respective disorders, and the clinical proforma was used to extract data from the case note. Those found with emotional disorders were psycho-educated and referred to the University of Maiduguri Teaching Hospital.

### Data analysis

The Statistical Package for Social Sciences software, version 20.0 (IBM [International Business Machines], Chicago, Illinois, United States-20), was used to analyse the data. The prevalence of depression and suicidality was determined using frequency and percentages. The Chi-square test was used to assess for an association between sociodemographic variables and clinical variables, depression and suicidality. Binary logistic regression was used to assess independent predictors of depression and suicidality. The level of statistical significance was set at *p* < 0.05.

### Ethical considerations

Ethical approval to conduct this study was obtained from the University of Maiduguri Teaching Hospital Maiduguri Ethical Review Board (No. FNPH/022023/REC154A) and the Neuro Psychiatric Hospital Research and Ethics Committee (No. NHREC/06/01/2020). Written informed consent was obtained from the study participants. To ensure confidentiality, codes were used for data entry and analysis. All questionnaires were anonymised. The children provided their assent, and the parents or other caregivers provided their formal consent after being informed.

## Results

Table 1 shows that 160 CALWHA, 77 (48.1%) were females and 83 (51.9%) were males. The participants’ ages ranged from 6 years to 18 years, with a mean age of 11.4 years (standard deviation [s.d.] = 3.15) and a median age of 11. More than half (52.5%) of the participants were between the ages of 10 years and 14 years. On educational attainment, 65.6% of the participants were in primary school, 45 participants (28.1%) were in secondary school and 10 participants (6.2%) were in tertiary institutions. About four-fifths of the participants (78.1%) were from small family sizes, and only 59.4% of the participants still have their parents alive. About 7.5% were not living with their parents. Most of the participants came from monogamous settings and small family sizes. The majority of the participants (80.0%) resided in urban areas, and 6.2% of respondents had a family history of mental illness. A prevalence rate of 45.6% was reported for depression, while a 30.6% prevalence rate was established for suicidality.

**TABLE 1:** Clinical and laboratory characteristics of participants (*N* = 160).

Variables	*n*	%
**No. of admissions**		
< 3 times	128	80.0
3	32	20.0
**Duration of treatment (years)**		
≤ 5	84	52.5
> 5	76	47.5
**Child awareness of disease**		
Yes	29	18.1
No	131	81.9
**WHO staging**		
I	149	93.1
II	7	4.4
III	4	2.5
**Type of ART**		
4d	160	100.0
Others	-	-
**Mode of transmission**		
Vertical	160	100.0
Horizontal	-	-
**Medication adherence**		
Yes	94	58.8
No	66	41.2
**CD4 count (cells/mm³)**		
< 500	10	6.3
≥ 500	150	93.7

ART, antiretroviral therapy; WHO, World Health Organization; 4d, Zidovudine, Lamivudine, Nevirapine; CD4, cluster of differentiation 4.

Majority (80%) of the participants had at least one admission during their illness. Only 58.8% of the participants adhered to their medication. Nearly all the participants (93.7%) had a CD4 count above 500 at the time of reporting the study, while only 6.3% recorded a CD4 count of less than 500 (Table 1).

Table 2 outlines the relationship between clinical and laboratory factors and depression. The number of previous admissions was found to have a statistically significant relationship with depression for participants with HIV (*p* = 0.003). Despite low numbers of non-adherent participants, the relationship between medication adherence and depression was still statistically significant (*p* = 0.001). The WHO staging was statistically significant for participants with HIV at a *p*-value of 0.007.

**TABLE 2:** Relationship between clinical and laboratory factors with depression.

HIV	Depression		No depression		Total		χ^2^	*p*
	*n*	%	*n*	%	*n*	%		
**No. of admissions**								
< 3 times	51	39.8	77	60.2	128	100	8.622	**0.003**
≥ 3	22	68.8	10	31.2	32	100	-	-
**Duration of treatment (years)**								
≤ 5	40	47.6	44	52.4	84	100	0.283	0.594
> 5	33	43.4	43	56.6	76	100	-	-
**WHO staging**								
I	73	49.0	76	51.0	149	100	9.911	**0.007**
II	-	-	7	100.0	7	100	-	-
III	-	-	4	100.0	4	100	-	-
**Type of ART**								
4d	73	45.6	87	54.4	160	100	-	-
Others	-	-	-	-	-	-	-	-
**Mode of transmission**								
Vertical	73	45.6	87	54.4	160	100	-	-
Horizontal	-	-	-	-	-	-	-	-
**Medication adherence**								
Yes	55	58.5	39	41.5	94	100	15.252	**0.001**
No	18	27.7	48	72.3	66	100	-	-
**CD4 count (cells/mm³)**								
< 500	3	30.0	7	70.0	10	100	1.050	0.306
≥ 500	70	46.7	80	53.3	150	100	-	-

Note: Bold values indicate *p*-value significance at < 0.05.

ART, antiretroviral therapy; WHO, World Health Organization; 4d, Zidovudine, Lamivudine, Nevirapine; CD4, cluster of differentiation 4.

Table 3 outlines the relationship between clinical and laboratory factors and suicidality. The number of previous admissions was found to have a statistically significant relationship with suicidal behaviour for participants with HIV (*p* = 0.001). Despite low numbers of non-adherent participants, the relationship between medication adherence and suicidal behaviour was significant (*p* = 0.001).

**TABLE 3:** Relationship between clinical and laboratory factors with suicidality.

HIV	Suicidality		No suicidality		Total		χ^2^	*p*
	*n*	%	*n*	%	*n*	%		
**No. of admissions**								
< 3 times	30	23.4	98	76.6	128	100	15.562	**0.001**
≥ 3	19	59.4	13	40.6	32	100	-	-
**Duration of treatment (years)**								
≤ 5	30	35.7	54	64.3	84	100	2.156	0.142
> 5	19	25.0	57	75.0	76	100	-	-
**WHO staging**								
I	49	32.9	100	67.1	149	100	5.214	0.074
II	-	-	7	100.0	7	100	-	-
III	-	-	4	100.0	4	100	-	-
**Type of ART**								
4d	49	30.6	111	69.4	160	100	-	-
Others	-	-	-	-	-	-	-	-
**Mode of transmission**								
Vertical	49	30.6	111	69.4	160	100	-	-
Horizontal	-	-	-	-	-	-	-	-
**Medication adherence**								
Yes	38	40.4	56	59.6	94	100	10.302	**0.001**
No	11	16.7	55	83.3	66	100	-	-
**CD4 count (cells/mm³)**								
< 500	2	50.0	2	50.0	4	100	0.725	0.395
≥ 500	47	30.1	109	69.9	156	100	-	-

Note: Bold values indicate *p*-value significance at < 0.05.

ART, antiretroviral therapy; WHO, World Health Organization; 4d, Zidovudine, Lamivudine, Nevirapine; CD4, cluster of differentiation 4.

### Multivariate logistic regression (binomial) for depression

Section A of Table 4 presents the significant findings of our study on the prevalence of depression among HIV participants. The multivariate logistic regression revealed that participants with three or more admissions were three times more likely to have depression (odds ratio [OR] = 3.3, confidence interval [CI] = 1.5–7.6) than those with fewer admissions. This prediction remained statistically significant even after adjusting for gender (OR = 3.3, CI = 1.4–7.5). Moreover, non-adherence to medications was associated with a fourfold increase in the likelihood of depression among participants with HIV (OR = 3.8, CI = 1.9–7.4). This prediction also remained statistically significant after adjusting for gender (OR = 3.7, CI = 1.9–7.4).

**TABLE 4:** Multivariate logistic regression (binomial) analysis.

Characteristics	Unadjusted OR (95% CI)	*p*	Adjusted OR (95% CI)	*p*
**(A) Depression in HIV patients**				
**Number of admissions**				
< 3	Ref	**0.0040**	Ref	**0.0050**
≥ 3	3.3 (1.5–7.6)		3.3 (1.4–7.5)†	
**Medication adherence**				
No	Ref	**< 0.0001**	Ref	**< 0.0001**
Yes	3.8 (1.9–7.4)		3.7 (1.9–7.4)†	
**(B) Suicidality in HIV patients**				
**Number of admissions**				
< 3	Ref	**< 0.0001**	Ref	**< 0.0001**
≥ 3	4.8 (2.1–10.8)		4.8 (2.1–10.9)†	
**Medication adherence**				
No	Ref	**0.0020**	Ref	**0.0020**
Yes	3.4 (1.6–7.3)		3.4 (1.6–7.3)†	

Note: Bold values indicate *p*-value significance at < 0.05.

OR, odds ratio; CI, confidence interval; HIV, human immunodeficiency virus; Ref, reference (base).

† , adjusted for gender.

### Multivariate logistic regression (binomial) for suicidality

Section B of Table 4 outlines the multiple logistic regression of HIV participants with suicidality. The multivariate logistic regression showed that three or more admissions had five times the likelihood of having suicidality among participants with HIV (OR = 4.8, CI = 2.1–10.8). This prediction remains statistically significant after adjusting for gender (OR = 4.8, CI = 2.1–10.9). Furthermore, non-adherence to medications was associated with three times the likelihood of having suicidality among participants with HIV (OR = 3.4, CI = 1.6–7.3). This prediction remains statistically significant after adjusting for gender (OR = 3.4, CI = 1.6–7.3).

## Discussion

The prevalence of depression among CALWHA was 45.6%. This finding aligned with a previous study that was conducted among people living with HIV and AIDS in Nigeria by Asogun and colleagues, who reported a prevalence rate of 66.7% for depression.^3^ However, this study was contrary to a similar study conducted in Lagos, Nigeria^1^ by Adeyemo et al. who reported a low prevalence rate (17.0%) for major depressive episodes among adolescents living with HIV. Differences in instrument sensitivity might be a reason for these discrepancies. For instance, Adeyemo et al. adopted the Mini International Neuropsychiatric Interview for Children and Adolescents (MINI-Kid). At the same time, this current study used the K-SADS-PL to assess psychiatric disorders such as depression and suicidality among the participants, ensuring a rigorous and comprehensive research process.

Similarly, suicidality prevalence was found to be 30.6%, which aligned with a previous study done by Adeyemo et al. (2020), where a prevalence rate of 35.0% was recorded for suicidality.^1^ However, the study disagreed with an Ethiopian study. Bogale et al. (2024) reported that suicidal ideation and attempts among HIV and AIDS patients in Ethiopia were 20.0% and 11.0%, respectively.^27^ This was lower than what was found in our study. The variations may be attributed to diverse demographic and cultural factors impacting the mental health of CALWHA across different regions. Other reasons for the higher prevalence of suicide among CALWHA in our study could be related to the Boko Haram insurgency and its resultant ripple effects, ranging from displacement to the deterioration of health indices in the region.

The study found a significant association between frequent hospital admissions and depression and suicidality among CALWHA. This finding underscores the importance of understanding the potential psychological distress that repeated hospitalisation may cause among CALWHA and their caregivers. Moreover, the study found a significant association between medication non-adherence, depression and suicidality. This further supports the link between ART non-adherence and mental well-being.

Regarding disease progression, most of the CALWHA in this study had CD4 counts above 500. Individuals with HIV who have a CD4 count greater than 500 are often in good health. The asymptomatic stage (I and II) was predominant, indicating a lower likelihood of emotional disorders compared to the symptomatic stage (III and IV). In all, 150 participants (93.7%) had a CD4 count above 500, while only 4 (6.3%) had a CD4 count less than 500. The association between low CD4 count and psychiatric complications has been widely reported.^18,19,20^ However, the fair adherence to the medication of 58.0% of HIV participants in our study might correspond with the majority of these participants being at the asymptomatic stage of the disease, thereby improving CD4 count and lowering the viral load. The asymptomatic WHO stages (I and II) are not positively related to having an emotional disorder, while the symptomatic stages (III and IV) have a higher likelihood of having a mental illness.^18,20^

All the participants are on the first-line combination of antiretroviral drugs termed 4d: Zidovudine, Lamivudine and Nevirapine (AZT-3TC-NVP). This may show the pattern of prescription and perceived efficacy by the attending paediatrician. In addition, studies have also shown an increased risk of developing emotional disorders such as depression with ART monotherapy (Zidovudine) and ART combination as well.^53^ In this study, the route of transmission of HIV was vertical for all the HIV participants (100%). A study found vertical transmission to be responsible for HIV infection in 90% of children.^54^ This could mean poor access to prevention of HIV from mother-to-child transmission (PMTCT) services, and coverage may be poor.

### Limitations

The study was cross-sectional, and causal relationships could not be established. Also, there may be a confounding effect of trauma as the insurgency has ravaged the study area for nearly 14 years. Lastly, this study was conducted in one of the teaching hospitals in northeastern Nigeria and only included 160 CALWHA. Given this, a generalised conclusion cannot be drawn to reflect the true situation of depression and suicidality among CALWHA.

## Conclusion

The observed prevalence rates of depression and suicidality, standing at 45.6% and 30.6%, respectively, underscore the significant burden of mental health challenges within this vulnerable population. The heightened risk of suicidality among CALWHA, coupled with its association with both hospitalisation and medication non-adherence, highlights the urgent need for targeted interventions to address the mental health vulnerabilities of this population. These interventions should extend beyond medical care to incorporate comprehensive psychosocial support, emphasising the importance of holistic approaches to enhance the overall well-being of CALWHA. It is therefore recommended that healthcare providers, policymakers and support organisations collaborate to develop integrated care strategies tailored to address the multifaceted mental health challenges among CALWHA. This comprehensive approach should not only encompass medical aspects but also prioritise the mental and emotional resilience of CALWHA, ultimately enhancing their overall quality of life.
